# Incidence of Atrial Fibrillation in Patients with either Heart Failure or Acute Myocardial Infarction and Left Ventricular Dysfunction: A Cohort Study

**DOI:** 10.1186/1471-2261-11-19

**Published:** 2011-05-14

**Authors:** Michelle D Schmiegelow, Ole D Pedersen, Lars Køber, Marie Seibæk, Steen Z Abildstrøm, Christian Torp-Pedersen

**Affiliations:** 1 Department of Cardiology, Gentofte University Hospital, Niels Andersens Vej 65, 2900 Hellerup, Denmark; 2 Department of Cardiology, Roskilde Sygehus, Køgevej 7-13, 4000 Roskilde, Denmark; 3 Department of Cardiology, The Heart Center, Rigshospitalet, Blegdamsvej 9, 2100 Copenhagen Ø, Denmark; 4 Department of Cardiology, Glostrup University Hospital, Nordre Ringvej 57, 2600 Glostrup, Denmark; 5 Department of Cardiology, Bispebjerg University Hospital, Bispebjerg Bakke, 2400 Copenhagen NV, Denmark

## Abstract

**Background:**

We examined the incidence of new-onset atrial fibrillation in patients with left ventricular dysfunction. Patients either had a recent myocardial infarction (with or without clinical heart failure) or symptomatic heart failure (without a recent MI). Patients were with and without treatment with the class III antiarrhythmic drug dofetilide over 36 months.

**Methods:**

The Danish Investigations of Arrhythmia and Mortality ON Dofetilide (DIAMOND) studies included 2627 patients without atrial fibrillation at baseline, who were randomised to treatment with either dofetilide or placebo.

**Results:**

The competing risk analyses estimated the cumulative incidences of atrial fibrillation during the 42 months of follow-up to be 9.6% in the placebo-treated heart failure-group, and 2.9% in the placebo-treated myocardial infarction-group.

Cox proportional hazard regression found a 42% significant reduction in the incidence of new-onset AF when assigned to dofetilide compared to placebo (hazard ratio 0.58, 95% confidence interval 0.40-0.82) and there was no interaction with study (p = 0.89).

In the heart failure-group, the incidence of atrial fibrillation was significantly reduced to 5.6% in the dofetilide-treated patients (hazard ratio 0.57, 95% confidence interval 0.38-0.86).

In the myocardial infarction-group the incidence of atrial fibrillation was reduced to 1.7% with the administration of dofetilide. This reduction was however not significant (hazard ratio 0.61, 95% confidence interval 0.30-1.24).

**Conclusion:**

In patients with left ventricular dysfunction the incidence of AF in 42 months was 9.6% in patients with heart failure and 2.9% in patients with a recent MI. Dofetilide significantly reduced the risk of developing atrial fibrillation compared to placebo in the entire study group and in the subgroup of patients with heart failure. The reduction in the subgroup with recent MI was not statistically significant, but the hazard ratio was similar to the hazard ratio for the heart failure patients, and there was no difference between the effect in the two studies (p = 0.89 for interaction).

## Background

Recent studies have indicated that particularly new-onset atrial fibrillation (AF) following hospitalization for heart failure or myocardial infarction (MI) is associated with a greater risk of death and stroke than permanent/persistent AF [1,2]. Although a number of studies have focused on AF in patients with heart failure and MI little is known about the incidence of new-onset AF following hospitalization in these populations [3,4]. Knowledge of this is important as it may influence the risk of thromboembolism and stroke and the potential benefit of antiarrhythmic treatment [5,6]. Due to lack of data on this matter, an expert panel appointed by The National Heart, Lung and Blood Institute has recently strongly recommended further research in AF prevention [7].

The Danish Investigations of Arrhythmia and Mortality ON Dofetilide (DIAMOND) studies randomised patients with left ventricular systolic dysfunction and either new or worsening heart failure or a recent MI to treatment with dofetilide or placebo.

We retrospectively analyzed data from the DIAMOND studies to investigate the incidence of AF in placebo-treated patients with left ventricular dysfunction and either heart failure or recent myocardial infarction. Secondarily we examined the potential benefit of treatment with dofetilide in these populations. Additionally, we assessed risk factors that may contribute to new-onset AF in these two cohorts in order to identify a population that could benefit from treatment with prophylactic anticoagulation or antiarrhythmic agents.

## Methods

We retrospectively analyzed data which was collected by the DIAMOND Investigators from November 1993 to November 1995 (DIAMOND-heart failure), and November 1993 to July 1996 (DIAMOND-MI). The DIAMOND investigations consisted of two separate, randomised, double-blind and multicentered studies. These investigated the safety and efficacy of the oral class III antiarrhythmic agent dofetilide in patients with left ventricular systolic dysfunction and either heart failure or a recent MI [8].

DIAMOND-heart failure enrolled 1518 patients with new or worsening heart failure corresponding to New York Heart Association functional class III or IV and left ventricular systolic dysfunction determined by echocardiography (left ventricular ejection fraction [LVEF] ≤ 35%) [9].

DIAMOND-MI enrolled 1510 patients who had experienced a MI within the last 7 days and had reduced left ventricular systolic dysfunction determined by echocardiography (LVEF ≤ 35%). The diagnosis required elevated enzymes and either typical chest pain and/or electrocardiographic changes suggestive of MI [10].

This study is based on the 12-lead electrocardiograms recorded during the first 72 hours of continuous monitoring at randomisation, and at each out-patient visit, which took place every 3^rd ^month. Patients with AF at the 12-lead electrocardiogram at randomisation were classified as baseline AF, and were excluded from the present analysis. Thus, new-onset AF was defined as patients having sinus rhythm at the 12-lead electrocardiograms recorded at randomisation and subsequently developed AF at the 12-lead electrocardiogram obtained at the out-patients visits. AF was defined as coarse or fine fibrillatory waves and completely irregular or regular RR-intervals. The diagnosis of AF was left to the discretion of the investigator [11].

The study complies with the Declaration of Helsinki [12]. It was approved by the Ethics Committee and all patients gave informed consent to participate in the study.

### Statistical Methods

Differences in baseline characteristics between groups were compared using the *χ^2 ^*procedure and the Mann-Whitney tests for categorical and continuous variables, respectively. Categorical variables are presented as counts and percentages. Continuous variables are presented as median values. All tests were two-sided. A p-value < 0.05 was considered significant.

The competing risk of death is important to take into account when examining the incidence of AF. Simple Kaplan-Meier curves essentially assume that the risk of AF is similar in patients remaining alive and those censored at the time of death. By using a competing risk model appropriate incidences of AF in patients remaining alive are calculated.

Predictors of new-onset AF were assessed by using Cox proportional hazard regression. The model assumptions (proportional hazard assumption, lack of interaction, and linearity of continuous variables) were tested and found valid unless otherwise indicated. In multivariable analyses we adjusted for heart failure, age, gender, diabetes, ischemic heart disease and LVEF. We furthermore tested, whether there was any interaction between drug and study, drug and age, and drug and gender in order to see whether the effect of dofetilide on new-onset AF differed according to study, age or gender, respectively.

The competing risk calculations were performed using Stata/IC 11.1 for Windows, whereas all other statistical calculations were performed using the SAS statistical software package for Windows users, version 9.1 (SAS Institute Inc, Cary, NC).

## Results

A total of 3028 patients with a LVEF ≤ 35% were enrolled in the two DIAMOND studies. For the purpose of this analysis 109 patients in the DIAMOND-MI study and 292 patients in the DIAMOND-heart failure study were excluded due to AF at baseline, leaving 2627 patients for further analysis. In the MI-study 693 patients (49.5%) were randomized to treatment with dofetilide and 708 patients (50.5%) to treatment with placebo. In the heart failure-group 626 patients (51%) were randomized to treatment with dofetilide and 600 patients (49%) to treatment with placebo.

A comparison of the baseline characteristics in patients who developed new-onset AF as opposed to those who did not is presented for each study in Table 1.

**Table 1:** Baseline characteristics of 1226 patients with heart failure and 1401 patients with a recent MI, respectively

	Baseline characteristics in DIAMOND-heart failure			Baseline characteristics in DIAMOND-MI		
	**Sinus rhythm** **(n = 1133)**	**New-onset AF** **(n = 93)**	**p-value**	**Sinus rhythm** **(n = 1369)**	**New-onset AF** **(n = 32)**	**p-value**

Age (mean)	69	71	0.12	68	71	0.07

Male gender	809 (71%)	79 (85%)	0.005	1011 (74%)	25 (78%)	0.59

Smoker	411 (36%)	24 (26%)	0.04	613 (45%)	14 (44%)	0.90

BMI	25.5	26.2	0.16	25.7	24.6	0.11

Previous MI	612 (54%)	49 (53%)	0.81	492 (36%)	11 (34%)	0.86

Diabetes	219 (19%)	23 (25%)	0.21	168 (12%)	3 (9%)	0.62

Angina pectoris	546 (48%)	35 (38%)	0.05	633 (46%)	15 (47%)	0.94

History of IHD	785 (69%)	63 (68%)	0.76	785 (57%)	18 (56%)	0.90

Hypertension	159 (14%)	15 (16%)	0.47	229 (17%)	5 (16%)	0.87

WMI	0.86	0.85	0.45	1.04	1.03	0.60

LVEF	0.26	0.25	0.45	0.31	0.31	0.60

Cr CL	56.4	55.0	0.56	64.7	68.0	<0.0001

ACE inhibitor	858 (76%)	73 (78%)	0.54	771 (56%)	13 (41%)	0.08

Digoxin	118 (10%)	6 (6%)	0.22	505 (37%)	7 (22%)	0.08

History of AF	71 (6%)	43 (46%)	<0.0001	23 (2%)	3 (9%)	0.001

ACE, angiotensin converting enzyme; AF, atrial fibrillation; BMI, body mass index; Cr CL, creatinine clearance; HF, heart failure; IHD, ischemic heart disease; LVEF, left ventricular ejection fraction; MI, myocardial infarction; WMI, wall motion index.

### Cumulative incidence of AF

**Figure 1:**
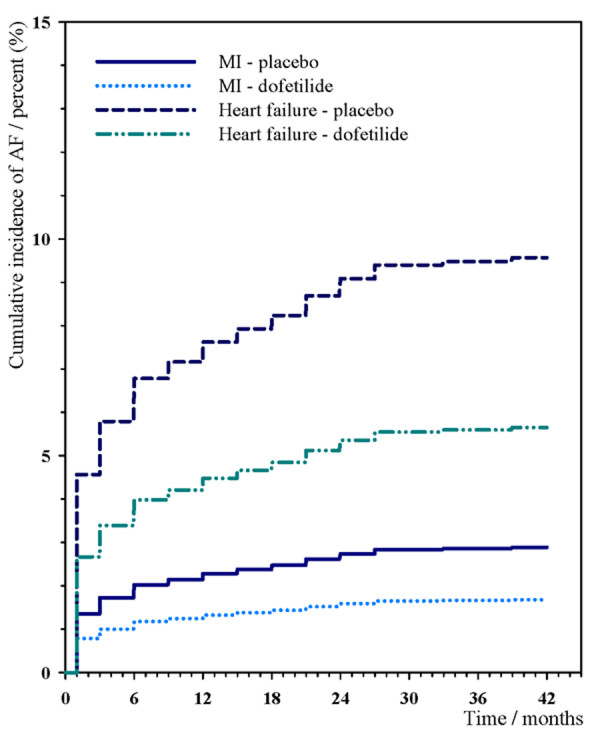
**The cumulative incidence of new-onset AF**. The figure shows the cumulative incidence of new-onset AF in patients with either MI or heart failure, and treated with either dofetilide or placebo. AF, atrial fibrillation; MI, myocardial infarction

Figure 1 shows the cumulative incidences of AF during the study period of 42 months. In the DIAMOND-heart failure study the cumulative incidences of new-onset AF in patients receiving placebo were estimated to be 6.8% after 6 months of follow-up, and 9.6% (n = 58) after 42 months of follow-up. The cumulative incidences were significantly reduced to 4.0% at 6 months of follow-up, and 6.8% (n = 35) in patients assigned to dofetilide (HR 0.57, 95%CI 0.38-0.86).

In the DIAMOND-MI study the incidence of new-onset AF in the placebo-treated patients was estimated to be 2.0% at 6 months of follow-up, and the cumulative incidence of new-onset AF in the 42-months of follow-up was 2.9% (n = 20). The risk reduction with the use of dofetilide was not significant in the MI-group. The incidences of AF in dofetilide-treated patients were estimated to be 1.2% at 6 months of follow-up and 1.7% (n = 12) after 42 months of follow-up (HR 0.61, 95%CI 0.30-1.24).

### Predictors of new-onset AF

In multivariable analyses we examined the risk factors of developing new-onset AF and adjusted for heart failure, age, gender, diabetes, ischemic heart disease and LVEF (Table 2). New-onset AF occurred more frequently in the heart failure group than in the MI group.

**Table 2:** Predictors of incident AF in patients with heart failure or a recent MI

	Hazard Ratio*	95%CI
Dofetilide compared to placebo	0.58	0.40-0.82

Heart failure	3.14	1.78-5.53

5-year age increase	1.17	1.06-1.28

Male gender	1.80	1.15-2.82

Diabetes	1.40	0.92-2.15

History of IHD	0.88	0.61-1.27

5-percentage increase in LVEF	0.87	0.77-0.99

AF, atrial fibrillation; CI, confidence interval; IHD, ischemic heart disease; LVEF, left ventricular ejection fraction; MI, myocardial infarction.

*Adjusted for heart failure, age, gender, diabetes, IHD and LVEF

We examined the predictors of new-onset AF in a single model, which included the entire population. We found an adjusted hazard ratio (HR) of 0.58 (95% confidence interval [CI] 0.40-0.82) corresponding to a 42% reduction in the incidence of new-onset AF when administering dofetilide compared to placebo. Furthermore, increasing age (HR 1.17 per 5-year age increase, 95%CI 1.06-1.29) and male gender (HR 2.01, 95%CI 1.25-3.24) were found to be significant predictors of new-onset AF. There was no interaction between drug and age (p = 0.26 for interaction) or between drug and gender (p = 0.80 for interaction).

The protective effect of increasing LVEF was significant with a relative risk reduction of 13% for each five percentage increase in LVEF (HR 0.87, 95%CI 0.77-0.99). Ischemic heart disease and diabetes were not found to predict new-onset AF. Heart failure was found to be the strongest predictor of new-onset AF (HR 3.14, 95%CI 1.78-5.52) in the combined analysis.

There was no interaction between drug and study (p = 0.89 for interaction).

## Discussion

This retrospective study explored the incidence of new-onset AF in patients with left ventricular systolic dysfunction and either heart failure or MI. We found that 1 in 10 patients with new or worsening heart failure and 1 in 35 patients with a recent MI developed new-onset AF during 42 months of follow-up. Dofetilide significantly reduced the incidence of AF with no interaction between drug and study.

Data from the Framingham Heart Study has found the lifetime risk of new-onset AF in the general population to be 1 in 4 in men and women from the age of 40-years and that new-onset AF is associated with a nearly 2-fold increased risk of death [13]. Nevertheless, a limited number of studies have investigated the incidence and risk of new-onset AF in populations with ischemic heart disease and heart failure. In this study, we examined the incidence of new-onset AF, whether AF was reduced by dofetilide and predictors of new-onset AF [3].

In our heart failure group (DIAMOND-heart failure) the cumulative incidence of new-onset AF was 9.6% (placebo group), and the cumulative incidence 6 months following hospitalization was 6.8%. Our long term result corresponds very well with the findings of recent studies. However we used a competing risk analysis to estimate cumulative risks which is in contrast to the use of the Kaplan-Meier estimator employed in other studies. As the populations being studied have a high mortality, in general, the results of other studies can be difficult to interpret as they study the cumulative incidence of AF in patients, without taking the competing risk of mortality of AF development into account. Simple Kaplan Meier graphs with censoring for death assumes a similar risk of AF in patients that die to those remaining in the analyses. This can cause distorted interpretations when the risk of death is high. Diversities between studies can also be accounted for by differences in study design and length of follow-up. The Prospective Randomized study of Ibopramine on Mortality and Efficacy (PRIME) II study enrolled patients with heart failure and left ventricular dysfunction with a mean follow-up of 4 years, during which the cumulative incidence of AF is 15% [14]. The retrospective study of the Valsartan Heart Failure Trial (Val-Heft) database examined the effect of the angiotensin-II receptor blocker valsartan on development of AF in patients with symptomatic heart failure and a LVEF ≤ 40% compared to placebo. During a mean follow-up of 23 months 8.0% in the placebo group and 5.1% in the valsartan group develop AF [15]. In the DIG trial, 8866 patients (11.1%) developed supraventricular tachycardia at least once during 37 months of follow-up. In the Carvedilol OR Metoprolol European Trial (COMET), 580 of 2429 (23%) develop AF during 58 months of follow-up.

The incidence of new-onset AF in patients with a recent MI has been the subject of few studies. In our MI group (DIAMOND-MI) the non-significant effect of dofetilide on the risk of developing AF can be explained by the few number of patients who experienced AF during follow-up (n = 32). Thus, this study statistically lacked power to provide any clear results of the association between MI and the effect of treatment with dofetilide on new-onset AF. However, we found no interaction with study that is the effect of dofetilide seemed to predict new-onset AF identically in the two populations.

The cumulative incidence of new-onset AF was 2.9% in the placebo group during 42 months of follow-up of which 2.0% happened during the first 6 months following the event. These results stand in contrast to the results of other studies, which can be explained by differences in important baseline characteristics such as hypertension and diabetes, as well as differences in the duration of follow-up. However, we cannot exclude that AF is underestimated in our study, as we did not examine for AF with continuous Holter monitoring.

In the Optimal Therapy In Myocardial infarction with the Angiotensin II Antagonist Losartan trial (OPTIMAAL) trial the cumulative incidence of new-onset AF was 7.2% in the study population of patients with MI and either clinical signs of heart failure or left ventricular dysfunction (LVEF≤40%) [1].

This corresponds to the finding in the GISSI-3 (Gruppo Italiano per lo Studio della Sopravvienza nell'infarto Miocardico-3) trial in which the overall incidence of in-hospital AF is 7.8% in the patients with a recent MI [16].

### Risk factors of new-onset AF

Because of the high risk of stroke and death associated with new-onset AF in heart failure and MI populations, prevention of new-onset AF following hospitalization for heart failure or MI may reduce mortality or stroke. Consequently, the identification of risk factors of the development of new-onset AF in patients hospitalized with heart failure or MI are of great importance.

In our analysis we found heart failure, increasing age, male gender and LVEF to predict new-onset AF. Heart failure was found to be the strongest predictor of new-onset AF as indicated by a number of other studies in which heart failure is found to be very common in patients with AF [1].

Increasing age and male gender are established risk factors of AF which correspond to our results and the effect of dofetilide did not depend on gender or age, as there was no interaction between drug and these parameters [1,14,15]. Furthermore, LVEF was confirmed as a predictor of new-onset AF which makes it easier to identify patients in high risk of developing AF in the daily clinical life, as the role of echocardiography is increasing.

The combination of these risk factors may be useful in upcoming trials testing prophylactic interventions against new-onset AF.

### The prognostic impact of AF

Reduction of the incidence of AF has not been shown to improve survival in patients with severe heart failure so far, nor has rhythm control been found to be superior to rate control in these patients [14,17]. Published data from the DIAMOND studies found no effect of dofetilide on the risk of mortality but hospitalization was reduced in the heart failure population [9,10]. Prevention of new-onset AF may, potentially, reduce morbidity and mortality because of the high risk of stroke and death associated with this condition. Interestingly, dronedarone treatment has recently been shown to reduce the primary endpoint of first cardiovascular hospitalization or death of any cause [18]. These results suggest that prevention of new-onset AF may have a favourable impact on outcome, but further studies are needed, especially in relation to patients who have had an MI.

## Limitations

This study has limitations that need to be specified. During the long-term follow-up of the DIAMOND-studies numerous treatment changes occurred, especially with regard to heart failure. These changes are impossible to adjust for in multivariable analysis. Moreover, the diagnosis of new-onset AF was based only on electrocardiograms recorded at outpatient visits. Therefore our findings are an underestimation of the events. On the other hand, many of the patients might have undiagnosed paroxysmal AF at baseline. However, the message of this paper remains unchanged; the relatively high incidence of AF should be kept in mind, when managing patients with left ventricular dysfunction and, especially, new or worsening heart failure. Furthermore, if symptomatic, rhythm control can be achieved effectively through prophylactic administration of dofetilide.

The DIAMOND-studies were not designed to evaluate the incidence of AF which might have contributed to some unknown biases, although the double-blinded randomization should have minimized biases. The size of this study was a limitation, especially due to the very high competing risk of mortality in the population. This was most notable in the MI-group, as the relatively few events in this group resulted in statistical lack of power to show a significant effect of dofetilide on the risk of new-onset AF.

## Conclusions

In patients with left ventricular dysfunction the incidence of AF at 42 months was 9.6% in patients with new or worsening heart failure and 2.9% in patients with a recent MI, and these results were based on a competing-risk analysis, that is, the estimated risk of developing AF, when the competing risk of death is taken into account.

The administration of dofetilide reduced the incidence of new-onset AF significantly by 42% compared to placebo and there was no interaction between dofetilide and study (p = 0.89 for interaction). Dofetilide is indicated as prophylactic antiarrhythmic treatment of AF in patients with left ventricular dysfunction, and either symptomatic heart failure or myocardial infarction.

## Competing interests

The authors declare that they have no competing interests.

## Authors' contributions

MDS (author of correspondence) analysed and interpreted the data, performed supplementary statistical analyses and drafted the manuscript. LK and MS assisted in the critical revision of the manuscript. ODP contributed to the interpretation of the data and the critical revision of the manuscript. SZA assisted in the statistical analyses and contributed to the critical revision of the manuscript. CTP performed the statistical analyses and contributed to the critical revision of the manuscript. LK and CTP participated in the design of the two DIAMOND-studies and participated together with MS in data collection. All authors have read and approved the final manuscript.

## Pre-publication history

The pre-publication history for this paper can be accessed here:

http://www.biomedcentral.com/1471-2261/11/19/prepub
